# Politeness

**Published:** 1876-05

**Authors:** 


### POLITENESS.

"How to Behave," is the title of a handsome thirty cent book of Fowler & Wells, which merits a very general circulation among the youth of our common country. It justly urges that the foundation of true politeness is in a kind heart and healthy body, that kindness is allied to intelligence, and that health is intimately associated with personal cleanliness, founding both propositions on the assertion, that "Cleanliness is akin to godliness." One of its sentiments, which, from its truthfulness, made a strong impression on our mind, was, "Unless children and youth are taught, by precept and example, to abhor what is selfish, and to prefer another's pleasure and comfort to their own, their politeness will be altogether artificial, and will be used only when interest and policy dictate." We have frequently noticed persons to be crusty, haughty, and unyielding, when they believed themselves unknown, but become all smiles and deference on the instant of recognizing some acquaintance of position.

